# A Beacon Interval Shifting Scheme for Interference Mitigation in Body Area Networks

**DOI:** 10.3390/s120810930

**Published:** 2012-08-07

**Authors:** Seungku Kim, Seokhwan Kim, Jin-Woo Kim, Doo-Seop Eom

**Affiliations:** Department of Electrical Engineering, Korea University, Seoul 136-713, Korea; E-Mails: kskkck@korea.ac.kr (S.K.); sukka@korea.ac.kr (S.K.); jjin300@korea.ac.kr (J.-W.K.)

**Keywords:** beacon interval shifting, body area network, IEEE 802.15.6, interference mitigation

## Abstract

This paper investigates the issue of interference avoidance in body area networks (BANs). IEEE 802.15 Task Group 6 presented several schemes to reduce such interference, but these schemes are still not proper solutions for BANs. We present a novel distributed TDMA-based beacon interval shifting scheme that reduces interference in the BANs. A design goal of the scheme is to avoid the wakeup period of each BAN coinciding with other networks by employing carrier sensing before a beacon transmission. We analyze the beacon interval shifting scheme and investigate the proper back-off length when the channel is busy. We compare the performance of the proposed scheme with the schemes presented in IEEE 802.15 Task Group 6 using an OMNeT++ simulation. The simulation results show that the proposed scheme has a lower packet loss, energy consumption, and delivery-latency than the schemes of IEEE 802.15 Task Group 6.

## 1. Introduction

Developments in wireless communication and the miniaturization of computing devices, such as wearable and implantable sensors, enable next-generation communication known as body area networks (BANs). Each BAN comprises several intelligent sensor nodes that should have a communication range of 3 m and dynamic data rates from 10 kbps to 10 Mbps according to application requirements [1,2]. Each sensor node monitors a human's biometric or surrounding environment information, and forwards it to a hub. Using this information, several applications can derive benefits from the BAN: healthcare, fitness, sports training, games, security, *etc.* IEEE 802.15 Task Group 6 (IEEE 802.15.6) has recently presented a draft [1] dealing with BANs.

BANs communicate around the human body, which is called intra-network communication. The BAN consists of one hub and sensor nodes. The hub coordinates the sensor nodes to avoid collisions, overhearing, or idle listening, which are associated with packet losses, energy consumption, and delivery-latency. Thus, the design of a medium access control (MAC) protocol is a challenging issue in BANs [3,4]. Although many MAC protocols have already been presented for wireless sensor networks (WSNs), they are inappropriate for BANs because of the unpredictable mobility patterns. The mobility of BANs leads to co-channel interference with other BANs or heterogeneous networks (e.g., WLAN, Bluetooth, and ZigBee in the ISM band). As a human moves, the BAN moves in and out of range of other networks. This mobility results in interference with other networks for a short time (e.g., walking or running on the street) or a long time (e.g., staying in a hospital or fitness center). Thus, it causes network-to-network interference [5] rather than node-to-node interference. Mitigating the network-to-network interference in BANs is very difficult due to absence of a central coordinator between the networks.

Conventional co-channel interference mitigation mechanisms have already been proposed in IEEE 802.15.2 [6] and Wireless Personal Area Networks (WPANs) [7]. In a WPAN, the basic interference mitigation mechanisms can be classified into three categories: space-division multiplexing (SDM), frequency-division multiplexing (FDM), or time-division multiplexing (TDM). In SDM, each network or node can share restricted space by adjusting its transmission power [8,9]. However, it could not completely eliminate interference from outside of the network because the human body absorbs radio signals and moves around. FDM allocates a dedicated frequency to each network in order to avoid interference. This technique wastes a great deal of time and resources in modifying the frequency. For this reason, it is unsuitable for the short-term interference environment of BANs. TDM is a method of sharing a limited time slot using a scheduling policy. In general, it is typically divided into a contention-based protocol, such as carrier sense multiple access (CSMA), and a schedule-based protocol, such as time division multiple access (TDMA). CSMA has low channel utilization because of the competition to obtain a shared medium. TDMA, in contrast, is difficult to schedule with heterogeneous networks.

In this paper, we propose a novel distributed TDMA-based beacon interval shifting scheme to reduce the BAN interference with other BANs or heterogeneous networks. A design goal of the proposed scheme is to avoid the wakeup period of each BAN coinciding with other networks. Each BAN hub performs carrier sensing on its channel before a beacon transmission. If the channel is idle, it transmits a beacon to reserve time for its network. Otherwise, the hub performs back-off for a defined period before operating the carrier sensing again. Through this process, the limited time can be allocated among various networks without a central coordinator in a distributed manner. We analyze the beacon interval shifting scheme and investigate the proper back-off length when the channel is busy. We perform simulations using the OMNeT++ simulator [10]. The simulation results show that the proposed scheme has a low packet loss, energy consumption, and delivery-latency compared with the schemes [1] presented in the IEEE 802.15.6 draft.

The rest of this paper is structured as follows: Section 2 discusses related work and introduces several problems in IEEE 802.15.6. We describe the beacon interval shifting scheme in Section 3 and evaluate the scheme in Section 4. Finally, Section 5 presents our conclusions.

## 2. Problem Definition and Related Works

In the near future, a number of persons will equip themselves with BANs around their body. In the areas of high population density such as hospitals and concert halls, the BAN on one person can experience interference from other co-located BANs. Cotton *et al.* [11] have conducted ray tracing simulations to compare the carrier-to-interference ratio for co-located BANs carried out at 2.45 GHz and 60 GHz. They showed carrier-to-interference ratio decreases in the simulation results as the number of co-located BANs increases.

**Figure 1.**
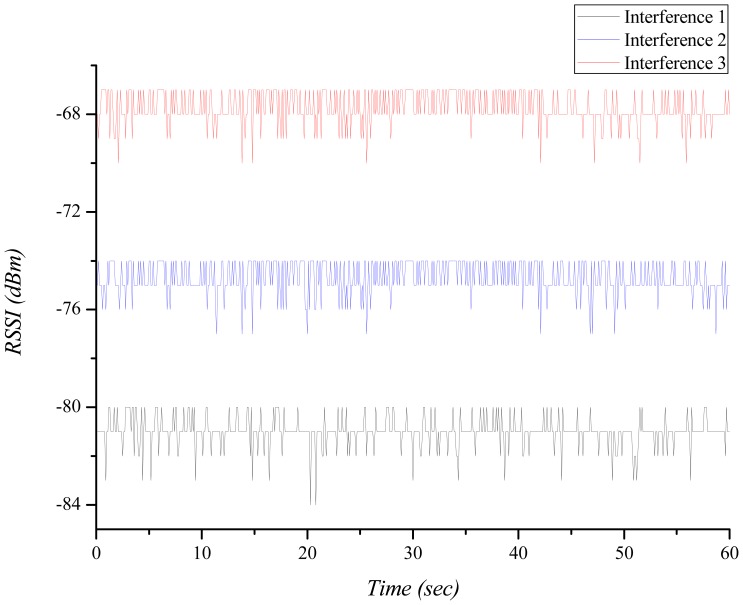
The measured RSSIs of the interference.

In order to investigate the interference effect on the performance of BANs, we conducted an empirical test. A BAN consists of a sender node (on wrist) and a receiver node (on pocket) on the person. The attached nodes are tmote sky [12] using the Chipcon CC2420 radio chip [13]. It operates in the 2.4 GHz. In this basic condition, we have 6 test scenarios. *Scenario 1*: A sender node continuously transmits carrier using CC2420 transmit test mode (duty cycle = 100%) with transmit power level 3. The distance between the sender node and receiver node is 0.5 m. *Scenario 2*: Based on the scenario 1, the distance between the sender node and receiver node is 1 m. *Scenario 3*: Based on the scenario 1, we added a barrier: a person's hand between the sender node and the receiver node. *Scenario 4*: Based on the scenario 1, we added another BAN that continuously generates interference using CC2420 transmit test mode (duty cycle = 100%) with transmit power level 7. The distance between two BANs is 4 m (Interference 1 in Figure 1). *Scenario 5*: Based on scenario 4, we changed the transmit power level to 11 (Interference 2 in Figure 1). *Scenario 6*: Based on scenario 4, we changed the distance between two BANs to 2 m (Interference 3 in Figure 1).

Figure 1 shows the received signal strength indication (RSSI) of the interference which is measured by the receiver node of BAN. It represents the degree of the interference. As the distance between two BANs approaches, the RSSI becomes higher and the interference effect increases.

**Figure 2.**
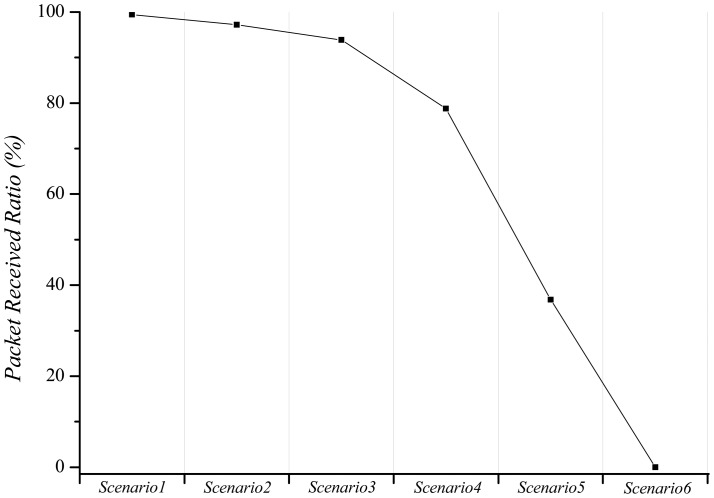
The measured PRR according to the different channel environment.

Figure 2 indicates the packet received ratio (PRR) in different scenarios. In scenario 1, scenario 2, and scenario 3, the packet loss gradually increases due to the signal attenuation. In scenario 4, scenario 5, and scenario 6, the BAN is influenced by the interference that is caused by another BAN. It depends on the distance between BANs and the transmit power level of the BAN. Because the mobility of persons varies the distance and the transmit power level, it has a possibility to cause a serious problem as if scenario 4, scenario 5, and scenario 6. Therefore, we should consider not only the signal attenuation effect but also the interference effect on the link in the BAN. In this paper, we focus on mitigating the interference between each BAN.

The common requirements in a BAN are to guarantee long network lifetime, short delays, and high reliability. MAC, transmission power control, and interference mitigation mechanisms are the main research areas attempting to satisfy the aforementioned requirements. A schedule-based MAC protocol such as TDMA allocates fixed or variable time slots to nodes, and they transmit independently using the assigned time slots. Therefore, TDMA does not encounter collisions, idle listening, or overhearing problems, and is considered to be a promising BAN MAC protocol [4].

The mobility and postural position of people incurs a co-channel interference that increases packet loss, resulting in energy waste and delivery-latency. This inter-network interference problem is difficult to solve due to the absence of a central coordinator. IEEE 802.15.6 has tried to reduce this inter-network interference. If a BAN exchanges its beacon in every fixed interval, the beacon and scheduled slots have a chance of repeated collisions with other networks using the same channel. Therefore, IEEE 802.15.6 proposed a beacon shifting scheme [1] in which a coordinator transmits its beacon in a different offset time relative to the beginning of the beacon period. Each beacon contains information of the next beacon period, and the offset time is decided by a beacon sequence that is not being used by neighbors. Through this process, it is expected that collisions with other networks will be reduced.

Another proposed scheme in IEEE 802.15.6 is frequency hopping. This is a typical interference mitigation scheme used in Bluetooth [14]. The basic concept is that a BAN periodically modifies its operating frequency for interference mitigation. In the IEEE 802.15.6 draft, a hub selects a frequency hopping sequence that is not being used by its neighbor, and delivers beacons including the next frequency hopping information to the sensor nodes. The BAN changes its operating frequency in the defined number of beacon periods. However, the different frequency hopping sequence also has the possibility of being overlapped by adjacent BANs because of the limited length of the frequency. Moreover, we cannot ignore frequency hopping overheads such as energy waste and delay.

In a static BAN environment, IEEE 802.15.6 performs energy detection or channel scanning [1] to seek an unoccupied channel before creating a new BAN. If there is no unoccupied channel, a coordinator requests time sharing with a BAN that already occupies a channel. These methods can benefit from static BANs. IEEE 802.15.6 proposes that a coordinator forces the sensor nodes to decrease their traffic or be disconnected according to their priority in the case that the packet error rate or received signal strength is below some fixed threshold [1]. This method will decrease network interference, but degrades channel utilization because generated traffic will be dropped by force.

## 3. The Beacon Interval Shifting Scheme

We assume that the proposed scheme is based on a TDMA-based MAC protocol. This is widely used for BAN MAC protocols to schedule the channel of a local network [4]. However, it is difficult to avoid interference from other networks using a TDMA-based MAC protocol because no central coordinator exists between the networks. In this paper, we present a novel distributed TDMA-based beacon interval shifting scheme to mitigate BAN interference with the other BAN or heterogeneous networks.

### 3.1. Description

In general, the TDMA-based MAC protocol periodically transmits a beacon that allocates dedicated time to each node and synchronizes the clock in the network. Thus, the beacon is a very important packet. Each network periodically wakes up and occupies the channel during its active period. Interference between the networks will occur if the occupied time of the channel overlaps with other networks. In cases where the nodes cannot receive the beacon successfully due to interference, they cannot use the channel for a whole beacon interval and will suffer long delays and waste energy. Therefore, we propose a TDMA-based beacon interval shifting scheme. The purpose of this scheme is to protect the beacon from interference and the active period overlapping with other networks.

**Figure 3.**
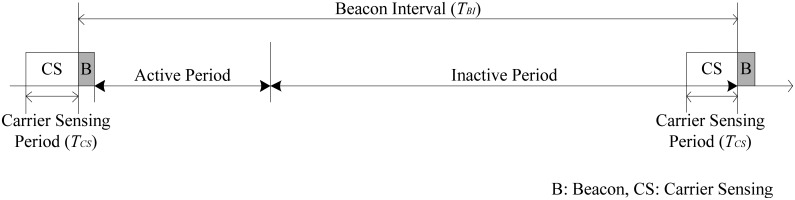
Superframe structure of the beacon interval shifting scheme.

Figure 3 illustrates the superframe structure of the beacon interval shifting scheme. Unlike conventional TDMA-based MAC protocols, a hub performs carrier sensing during a fixed period, *T_CS_*, before its beacon transmission. The carrier sensing period can be decided independently according to the application requirements of each BAN. During the carrier sensing period, the hub identifies whether the channel is occupied by other networks or not. If the channel is idle during the carrier sensing period, the hub transmits a beacon immediately. As the hub only performs carrier sensing before a beacon transmission, this technique does not provide any overhead to the nodes when the channel is in an idle state.

**Figure 4.**
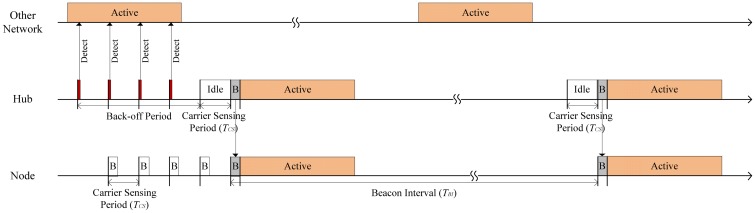
A sample of the beacon interval shifting scheme.

Figure 4 shows an operating sample of the beacon interval shifting scheme. If the channel is busy, the hub does not transmit a beacon and performs a back-off procedure until the channel becomes idle. As shown in Figure 4, the hub delays its beacon transmission until the next carrier sensing period, *T_CS_*, when it detects that the channel is in a busy state. The hub repeats the carrier sensing until the channel is idle during the whole carrier sensing period. We call the repeated carrier sensing periods a back-off period in this paper. At the same time, the node periodically wakes up and identifies a beacon transmission in every carrier sensing period. If the node did not identify a beacon transmission, it immediately sleeps. Thus, it wastes a little energy even if a lot of back-offs occur. If the channel becomes idle, the nodes can safely receive the beacon from the hub. Through this procedure, the beacon interval of the BAN moves back by the back-off period, so the active period of each network is uniformly arranged in the same channel. Hence, the network does not need to back-off again in the next beacon period. By means of this back-off, we can reduce the interference between networks.

The back-off period can be long if many networks attempt to use the same channel simultaneously. Therefore, our scheme modifies the current channel when the back-off period exceeds a pre-determined threshold. This threshold should not be higher than the delivery-latency demanded by the application. This channel modification can decrease the possibility of a lot of networks converging on the same channel.

### 3.2. Analysis According to the Length of the Carrier Sensing Period

In the beacon interval shifting scheme, the hub performs its carrier sensing before a beacon transmission. The performance of the scheme depends on the length of the carrier sensing period. In this Section, we analyze the probability of a channel being busy, the energy consumption, and the average delivery-latency according to various carrier sensing periods.

The number of events in any interval *t* is the Poisson distribution with mean *λ·t*. Hence, the probability of *k* event occurrences in the interval *t* is given by:

(1) $$P\left(k,t\right)=\frac{{\left(\lambda \cdot t\right)}^{k}}{k!}\cdot e^{-\lambda \cdot t}$$

where *λ* is the expected number of event occurrences during the given interval.

**Figure 5.**
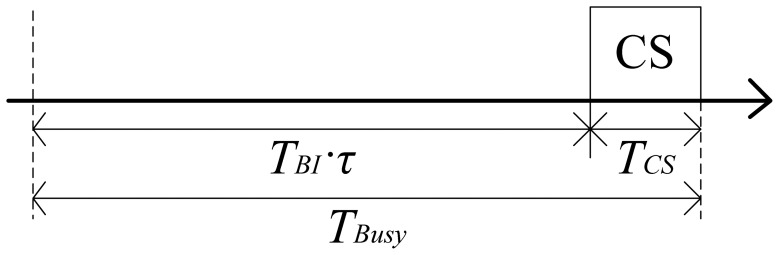
Possible channel busy period.

We assume that each network occupies the channel for the successive time as long as the duty cycle *τ*. The channel holding time of the BAN is thus identical to the active period *T_BI_·τ* in a beacon interval. The hub judges the channel state during its carrier sensing period. In order to be in the busy state, the active period of the other networks has to overlap with its carrier sensing period, *T_CS_*. The hub decides that the channel is in the busy state if the active period of the other networks begins in the possible channel busy period, denoted by *T_Busy_* in Figure 5.

In the beacon interval shifting scheme, the active periods do not overlap with those of other networks because of the back-off performed after carrier sensing. Therefore, the average occurrence rate of the active period in a beacon interval is as follows:

(2) $$\lambda =\frac{1}{T_{\mathrm{BI}}}+\frac{1}{T_{\mathrm{BI}}-T_{\mathrm{BI}}\cdot \tau }+\frac{1}{T_{\mathrm{BI}}-2\cdot T_{\mathrm{BI}}\cdot \tau }+\cdots +\frac{1}{T_{\mathrm{BI}}-\left(n-1\right)\cdot T_{\mathrm{BI}}\cdot \tau }=\sum_{i=1}^{n}\frac{1}{T_{\mathrm{BI}}-\left(i-1\right)\cdot T_{\mathrm{BI}}\cdot \tau }$$

where *n* is the number of networks in the co-channel.

By using Equations (1) and (2), we can obtain the idle probability of the channel as follows:

(3) $$P_{\text{Idle}}=P\left(0,T_{\text{Busy}}\right)=e^{-\lambda \cdot T_{\text{Busy}}}$$

and the busy probability of the channel can be defined as:

(4) $$P_{\text{Busy}}=1-P\left(0,T_{\text{Busy}}\right)=1-e^{-\lambda \cdot T_{\text{Busy}}}$$

**Figure 6.**
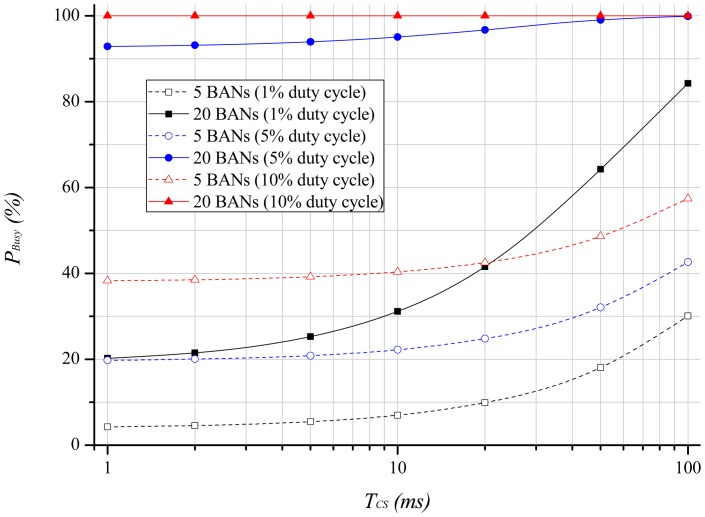
Channel busy probability according to carrier sensing period.

Figure 6 shows the channel busy probability as the length of the carrier sensing period increases. We assume that the beacon interval is 1.28 s. As we can infer from the previous equations, many BANs and a high duty-cycle lead to a long channel holding time that results in a high probability of the channel being busy. This probability, furthermore, increases as the carrier sensing period, *T_CS_*, becomes longer. This is because *T_Busy_* becomes longer as *T_CS_* increases.

Most BAN environments assume that the hub has sufficient resources, such as battery and memory. However, as the nodes suffer from resource constraints, energy-efficiency is also an important factor. If the channel is already being used by other networks, the hub and nodes should back-off. At this moment, the nodes cannot receive a beacon even if they enter Rx mode during the beacon period, which causes extra energy waste. The extra energy waste at each node can be calculated by:

(5) $$\begin{array}{cc} E_{\text{Node}} & =T_{\text{Beacon}}\cdot PW_{\mathrm{Rx}}\cdot P_{\text{Busy}}\cdot P_{\text{Idle}}+2\cdot T_{\text{Beacon}}\cdot PW_{\mathrm{Rx}}\cdot {\left(P_{\text{Busy}}\right)}^{2}\cdot P_{\text{Idle}}+3\cdot T_{\text{Beacon}}\cdot PW_{\mathrm{Rx}}\cdot {\left(P_{\text{Busy}}\right)}^{3}\cdot P_{\text{Idle}}+\cdots +m\cdot T_{\text{Beacon}}\cdot PW_{\mathrm{Rx}}\cdot {\left(P_{\text{Busy}}\right)}^{m}\\ & =T_{\text{Beacon}}\cdot PW_{\mathrm{Rx}}\cdot \left\{\sum_{k=1}^{m-1}k\cdot {\left(P_{\text{Busy}}\right)}^{k}\cdot P_{\text{Idle}}+m\cdot {\left(P_{\text{Busy}}\right)}^{m}\right\} \end{array}$$

where *T_Beacon_* is the time taken to receive a beacon, *PW_Rx_* is the power consumption in Rx mode, and *m* represents the maximum number of back-offs. In other words, the network modifies its current channel after *m* back-offs. We should determine *m* such that the maximum back-off period, *m*·*T_CS_*, is below the delivery-latency required by the application.

**Figure 7.**
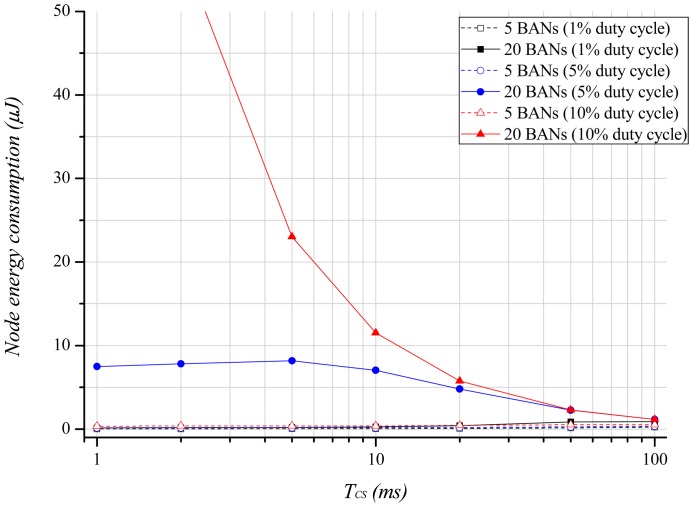
Node energy consumption according to the carrier sensing period.

Figure 7 illustrates the energy consumption of a node with regard to the carrier sensing period. We assume that *PW_Rx_* is 2.9 mW, and the maximum delivery-latency demanded by the application is 200 ms in this analysis. As Figure 6 shows, the increase of BANs and a high duty-cycle result in a high probability of the channel being busy, which causes repeated back-offs. Thus, the energy consumption is high with many BANs and a high duty-cycle. As the carrier sensing period becomes longer, the energy consumption decreases because the long carrier sensing period increases the wakeup interval for beacon reception. Therefore, an application demanding energy-efficiency can take advantage by using a longer carrier sensing period.

The delivery-latency is as important as the energy-efficiency in a BAN. If the network successively backs-off due to the channel being busy, the delivery-latency increases. In order to observe the relationship between the carrier sensing period and the delivery-latency, we consider the following Equation:

(6) $$\begin{array}{cc} \mathrm{DL} & =T_{\mathrm{CS}}\cdot P_{\text{Busy}}\cdot P_{\text{Idle}}+2\cdot T_{\mathrm{CS}}\cdot {\left(P_{\text{Busy}}\right)}^{2}\cdot P_{\text{Idle}}+3\cdot T_{\mathrm{CS}}\cdot {\left(P_{\text{Busy}}\right)}^{3}\cdot P_{\text{Idle}}+\cdots +\left(m-1\right)\cdot T_{\mathrm{CS}}\cdot {\left(P_{\text{Busy}}\right)}^{m-1}\cdot P_{\text{Idle}}+m\cdot T_{\mathrm{CS}}\cdot {\left(P_{\text{Busy}}\right)}^{m}\\ & =T_{\mathrm{CS}}\cdot \left\{\sum_{k=1}^{m-1}k\cdot {\left(P_{\text{Busy}}\right)}^{k}\cdot P_{\text{Idle}}+m\cdot {\left(P_{\text{Busy}}\right)}^{m}\right\} \end{array}$$

Equation (6) has the same back-off probability as Equation (5), except that the energy consumption has been replaced by the carrier sensing period.

**Figure 8.**
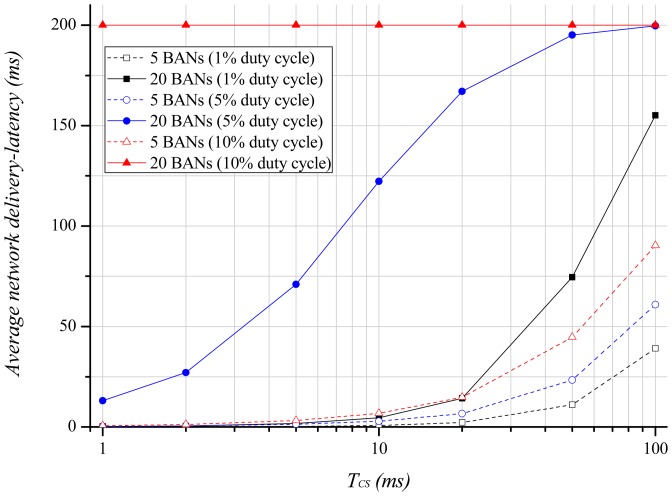
Average network delivery-latency according to the carrier sensing period.

Figure 8 describes the average network delivery-latency with regard to the length of the carrier sensing period. Here, the delivery-latency is the data delivery time from the node to the hub or from the hub to the node. The average delivery-latency of 20 BANs and a 10% duty-cycle exhibits the maximum delivery-latency allowed by the application because the channel is always in the busy state. This means that a longer channel holding time increases the average delivery-latency. However, contrary to the energy consumption results, a longer carrier sensing period leads to a higher average delivery-latency because the length of the back-off is extended. Therefore, we have to adjust the length of the carrier sensing period according to the application requirement, as there is a tradeoff between the delivery-latency and energy consumption.

## 4. Performance Evaluation

In this Section, we compare the performance of the beacon interval shifting scheme with IEEE 802.15.6. The OMNeT++ [8] simulator is used to evaluate the performance of each protocol. Because BANs attached to people can be mobile or remain stationary, we simulated both scenarios: the stationary case and the mobile case.

### 4.1. Parameter Set

We use an OMNeT++-based Castalia simulation model [15] that implements the IEEE 802.15.6 draft. Each network consists of one hub and four nodes in star topology. In this simulation model, the characteristics of the radio transceiver follow BANRadio [15], which is designed according to IEEE 802.15.6 requirements. The BAN Radio has a 1,024 Kbps data rate, −104 dBm noise floor, and −87 dBm sensitivity. Each node uses 3.1 mW in Rx mode and 2.9 mW in Tx mode (−20 dBm). The path loss model [16] according to the distance *d* in free space is as follows:

(7) $$\mathrm{PL}(d)=PL_{0}+10\cdot n\cdot \log_{10}\left(\frac{d}{d_{0}}\right)+S$$

where *PL_0_* is the path loss coefficient at a reference distance *d0*, and *n* is the path loss exponent. S represents the shadowing component that is a Gaussian zero-mean random variable with standard deviation σ. We basically set *PL_0_* = 52, *d_0_* = 1, *n* = 2.4, and σ = 4, but *PL_0_* between a hub and a node takes a random value from 55 to 60 due to the dynamic postural position of the human body. We assume that the beacon interval is 1.28 s and the duty-cycle of each network is 10%. The carrier sensing period, *T_CS_*, in the beacon interval shifting scheme is 10 ms that showed low energy consumption and network delivery-latency in the analysis. In the case of a mobile BAN, we assume random movement in a 100 m × 100 m field at 1.39 m/s (*i.e.*, the average walking speed of a human). In order to evaluate the results according to the number of BANs, we do not set a maximum value of *m*, the number of back-offs, which prohibits the channel modification.

### 4.2. Evaluation Results in the Stationary Case

In this subsection, we evaluate the performance of a BAN in the stationary case. We assume that BANs cause interference with each other within their interference range, so the BAN is influenced by long-term interference in the stationary case. In the results, we refer to IEEE 802.15.6 without any interference mitigation schemes as the basic scheme, and assume that the beacon shifting scheme and the beacon interval shifting scheme operate on IEEE 802.15.6.

**Figure 9.**
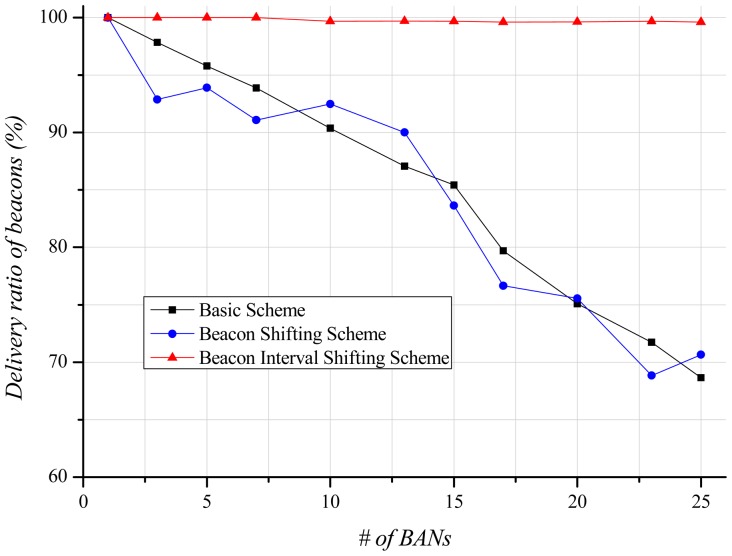
Delivery ratio of beacons according to the number of BANs.

A beacon contains limited information (e.g., beacon interval, time slot scheduling information, timestamp). The beacon is an important packet as it schedules time slots for each node. If a node fails to receive the beacon, it cannot transmit and receive until the next beacon interval. This influences the delivery ratio, and is thus closely related to energy consumption and delivery-latency. Figure 9 shows the delivery ratio of beacons according to the number of BANs. By increasing the number of BANs, the basic scheme indicates a lower delivery ratio of beacons due to high interference in the channel. If the beacon shifting scheme is used, each BAN transmits beacons according to its unique beacon sequence index. Thus, IEEE 802.15.6 expects to avoid repeated beacon collisions with its neighbor BANs. Figure 9, however, shows similar results to the basic scheme. Since there is no central coordinator among BANs, interference and collision still occur randomly even if each BAN uses the unique beacon sequence index. Our beacon interval shifting scheme displays almost a 100% beacon delivery ratio. Carrier sensing before beacon transmission enables this scheme to identify the channel state, and, thus, each BAN can avoid beacon collisions with other BANs.

**Figure 10.**
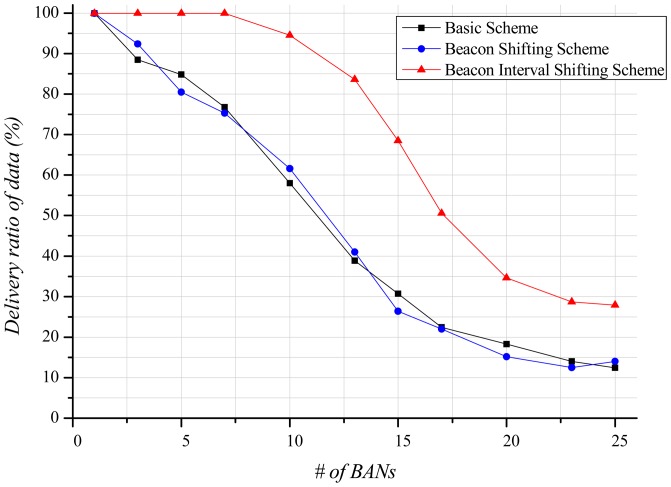
Delivery ratio of data according to the number of BANs.

A node cannot use the channel until it successfully receives a beacon. Therefore, the delivery ratio of data is closely related to the delivery ratio of beacons. Figure 10 shows that, as the number of BANs increases, the delivery ratio of data decreases. In the basic scheme and the beacon shifting scheme, the main reason for this decrease is packet loss due to active time overlaps among BANs. Moreover, in cases of beacon reception failure and packet losses, the retransmission of data increases. These retransmissions lead to a higher probability of buffer overflow, resulting in packet drops. In contrast, the beacon interval shifting scheme hardly has any packet losses as it guarantees an idle channel by performing carrier sensing before beacon transmission. Nevertheless, the delivery ratio of data declines as the number of BANs increases. A BAN with many neighbor BANs has a higher probability of encountering a busy channel, and extends its back-off time to occupy the channel. The extended back-off time causes frequent buffer overflows, resulting in many packet drops. As shown in Figure 10, the delivery ratio of data gradually decreases in the beacon interval shifting scheme. It does, however, show an approximately 35% higher delivery ratio than the other schemes because it has lower packet loss.

**Figure 11.**
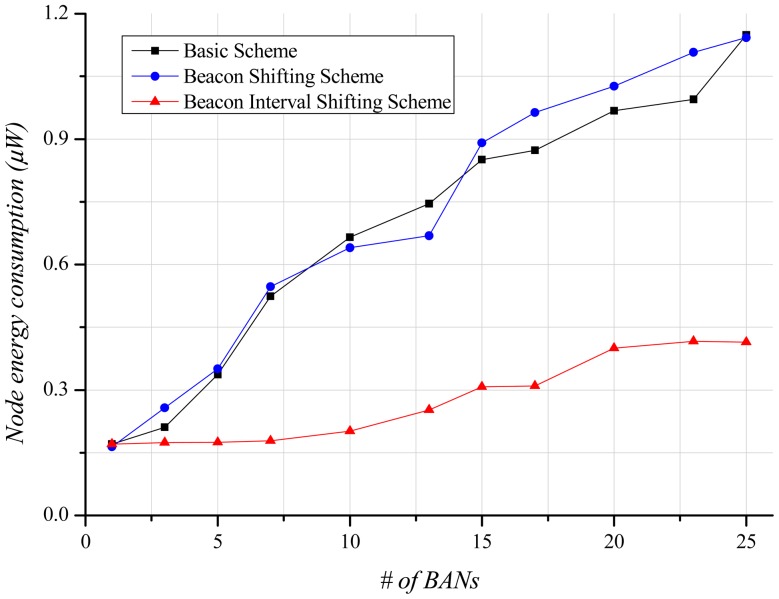
Node energy consumption according to the number of BANs.

Energy-efficiency is an important factor in BANs. A node has less battery power than a hub. Accordingly, the energy-efficiency of a node is more important than that of the hub. Figure 11 illustrates the steep growth in energy consumption of the basic scheme and the beacon shifting scheme as the number of BANs increases, because many missed data are continuously retransmitted regardless of transmission success or failure. The beacon interval shifting scheme consumes less energy than the other schemes because data retransmissions owing to packet losses scarcely occur. However, a lot of back-offs lead the nodes to needlessly overhear the channel for beacon receptions. For that reason, energy consumption also increases with the number of BANs, as shown in Figure 11.

**Figure 12.**
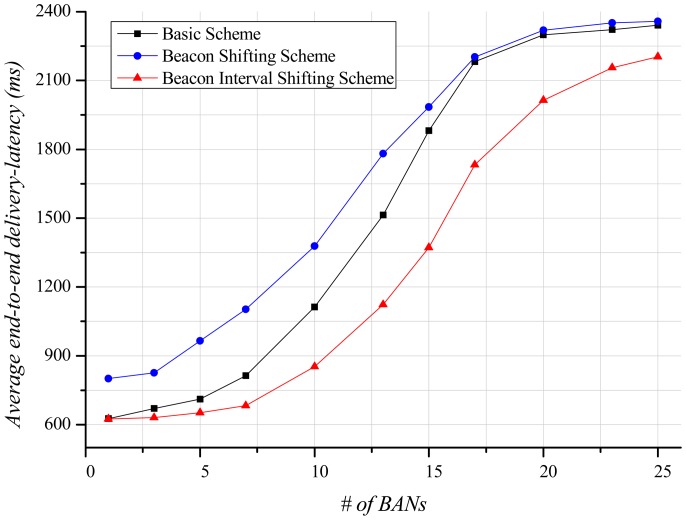
Average end-to-end delivery-latency according to the number of BANs.

Delivery-latency is also an important factor determining the performance of a BAN. In this paper, the end-to-end delivery-latency indicates the duration from the occurrence time of application data at a sender to the arrival time of the data at a receiver. This is different meaning from the network delivery-latency in Figure 8. Figure 12 indicates the average end-to-end delivery-latency according to the number of BANs. In case of one BAN in the field, the average end-to-end delivery-latency of the basic scheme and our beacon interval shifting scheme are 625.95 ms and 625.14 ms, respectively. This is due to no interference from other BANs. The beacon shifting scheme, on the other hand, has variable beacon intervals from 320 ms to 2,240 ms according to the beacon sequence index. Because of these variable beacon intervals, its average end-to-end delivery-latency is 800.96 ms, which is longer than the other schemes. As the increase of BANs, the interference increases, and the delivery ratio of data decreases. It causes more retransmissions and buffered data that result in a longer average end-to-end delivery-latency. Therefore, the beacon interval shifting scheme has fewer retransmissions due to packet losses. Thus, it can deliver more data than the other schemes and has a lower average end-to-end delivery-latency in Figure 12.

### 4.3. Evaluation Results in the Mobile Case

In this subsection, we evaluate the performance of a BAN in the mobile case. As each BAN moves randomly and causes interference with other BANs, the mobile case represents short-term interference. BANs randomly move at the average walking speed of a human in a 100 m × 100 m field, and interfere with each other.

**Figure 13.**
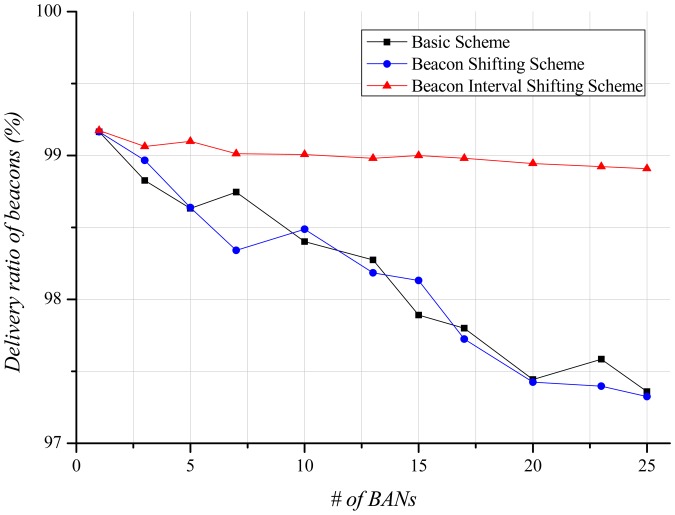
Delivery ratio of beacons according to the number of BANs.

Figure 13 illustrates the delivery ratio of beacons depending on the number of BANs. As for the stationary case, the delivery ratio of beacons decreases as more BANs exist in the field. However, because of its local mobility within the BAN, even when one BAN exists in the field a few beacons are missed, resulting in about a 99% beacon delivery ratio. In the basic scheme and the beacon shifting scheme, the delivery ratio of beacons gradually decrease. It is marginal improvement compared with the stationary case because the interference effect is too low. However, the beacon is a very important packet. If a beacon fails to be delivered, the BAN cannot deliver data during the entire superframe since the time slots are not scheduled. Thus, the delivery ratio of data is highly dependent on the delivery ratio of beacon. The channel state obtained by carrier sensing is not perfectly accurate because the mobility of BANs continuously changes the channel state. For this reason, the delivery ratio of beacons decreases slightly in the beacon interval shifting scheme.

**Figure 14.**
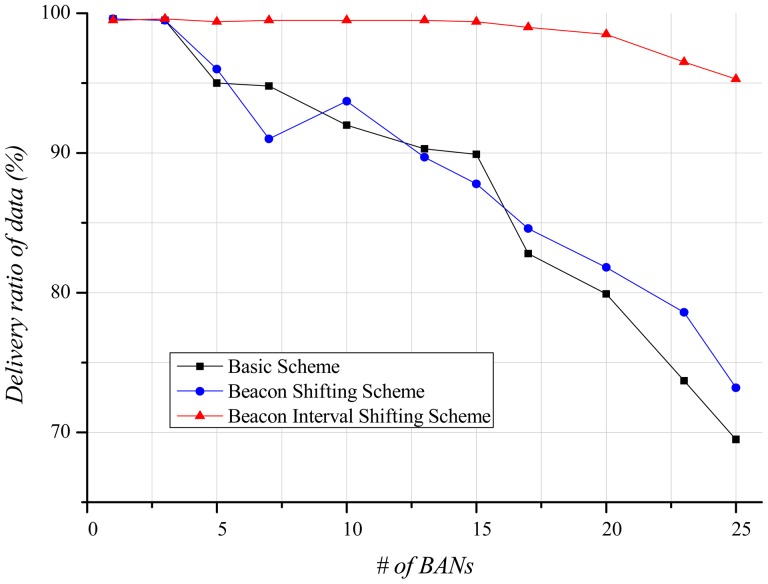
Delivery ratio of data according to the number of BANs.

As shown in Figure 14, the delivery ratio of data exhibits a similar form as that of the delivery ratio of beacons in the mobile case. The delivery ratio of data in the basic scheme and the beacon shifting scheme falls as the number of BANs increases. The slope, however, is less steep than in the stationary case because the mobility of the BANs leads to less interference with each other, and the reduced interference decreases the number of packet losses and buffer overflow. The delivery ratio of the beacon interval shifting scheme gradually decreases as the number of BANs increases. Unlike the stationary case, buffer overflow seldom occurs because of the reduced interference. However, our scheme experiences some packet losses, which rarely happen in the stationary case. In the stationary case, packet losses can be avoided by performing carrier sensing before the beacon transmission. However, in the mobile case, the active period of BANs may partially overlap because the channel state is continuously changing. This degrades the delivery ratio of data, but the beacon interval shifting scheme still shows better performance than the other schemes.

**Figure 15.**
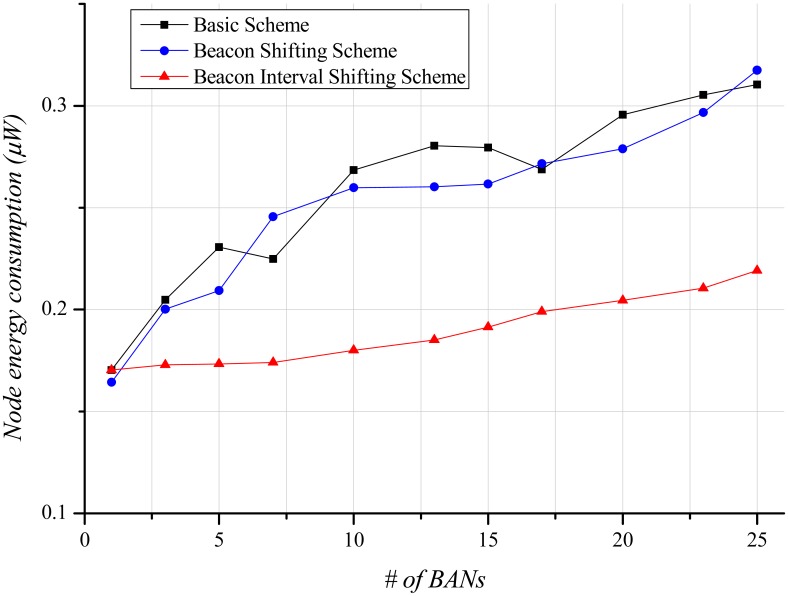
Node energy consumption according to the number of BANs.

As the number of BANs increases, the energy consumption of a node steadily grows (Figure 15) because of the increase of retransmissions. However, in comparison with the stationary case, the slope of the energy consumption is gentler because of fewer packet losses and less buffer overflow. Moreover, the beacon interval shifting scheme performs fewer back-offs. This enables the node to reduce beacon overhearing, thus reducing energy waste.

**Figure 16.**
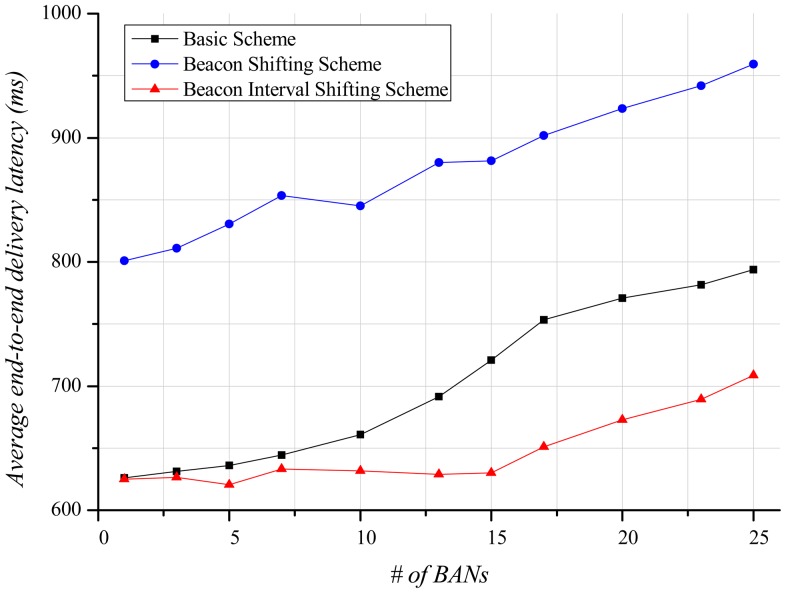
Average end-to-end delivery-latency according to the number of BANs.

Figure 16 displays the average end-to-end delivery-latency according to the number of BANs. When only one BAN exists, the mobile case gives almost the same result as shown in Figure 12, since interference does not exist. As the number of BANs increases, the average end-to-end delivery-latency increases like the stationary case. However, a slope of the schemes indicates gentler than the stationary case because of less interference in the mobile case. The beacon interval shifting scheme shows low average end-to-end delivery-latency because it experiences less interference. The beacon shifting scheme still presents a higher average end-to-end delivery-latency due to its variable beacon intervals.

## 5. Conclusions

In this paper, we presented a beacon interval shifting scheme that performs carrier sensing before beacon transmission. Using this carrier sensing, the active period of BANs could be automatically arranged. We simulated the basic scheme, the beacon shifting scheme, and the beacon interval shifting scheme based on the IEEE 802.15.6 draft. In contrast to our expectations, the basic scheme and the beacon shifting scheme showed similar results for delivery-ratio and energy consumption. The beacon shifting scheme even had a longer delivery-latency than the basic scheme. The interference from other networks is randomly distributed in the channel, so beacon shifting is useless for avoiding interference. Delivery ratio and energy consumption in the beacon interval shifting scheme showed better performance than the other schemes in both the stationary case and the mobile case. In particular, our scheme exhibited much better performance in the stationary case because the channel state is not frequently changed, as in the mobile case. Although we only simulated BAN to BAN interference, our scheme can also be applicable to avoid interference from heterogeneous networks by deferring the beacon interval or switching the current channel. It can be conducted without any protocol changes of the heterogeneous network and any interactions between the BAN and the heterogeneous network. Even though the back-off before beacon transmission is not allowed by FCC regulations so far, we expect that the beacon interval shifting scheme will be a practical solution for interference mitigation in case that the regulations change.
